# Improved ChIP-chip analysis by a mixture model approach

**DOI:** 10.1186/1471-2105-10-173

**Published:** 2009-06-07

**Authors:** Wei Sun, Michael J Buck, Mukund Patel, Ian J Davis

**Affiliations:** 1 Department of Biostatistics, Carolina Center for Genome Sciences, University of North Carolina at Chapel Hill, Chapel Hill, NC, USA; 2 Department of Biochemistry, Center of Excellence in Bioinformatics and Life Sciences, State University of New York at Buffalo, Buffalo, NY, USA; 3 Department of Genetics, University of North Carolina at Chapel Hill, Chapel Hill, NC, USA; 4 Department of Pediatrics, Lineberger Comprehensive Cancer Center, University of North Carolina at Chapel Hill, Chapel Hill, NC, USA

## Abstract

**Background:**

Microarray analysis of immunoprecipitated chromatin (ChIP-chip) has evolved from a novel technique to a standard approach for the systematic study of protein-DNA interactions. In ChIP-chip, sites of protein-DNA interactions are identified by signals from the hybridization of selected DNA to tiled oligomers and are graphically represented as peaks. Most existing methods were designed for the identification of relatively sparse peaks, in the presence of replicates.

**Results:**

We propose a data normalization method and a statistical method for peak identification from ChIP-chip data based on a mixture model approach. In contrast to many existing methods, including methods that also employ mixture model approaches, our method is more flexible by imposing less restrictive assumptions and allowing a relatively large proportion of peak regions. In addition, our method does not require experimental replicates and is computationally efficient. We compared the performance of our method with several representative existing methods on three datasets, including a spike-in dataset. These comparisons demonstrate that our approach is more robust and has comparable or higher power than the other methods, especially in the context of abundant peak regions.

**Conclusion:**

Our data normalization and peak detection methods have improved performance to detect peak regions in ChIP-chip data.

## Background

Microarray based analysis of immunoprecipitated chromatin (ChIP-chip) constitutes a powerful technique to detect the interaction of DNA with regulatory proteins over large segments of chromatin [1,2]. With advances in microarray fabrication, high-density tiling arrays are now being employed for genome-wide ChIP-chip studies [3,4]. In ChIP-chip, immunoprecipitated chromatin is amplified, fluorescently labeled and hybridized to a tiled DNA microarray. Fluorescent signal detected from hybridization to several oligomers representing a contiguous region is graphically depicted as a "peak" and is suggestive of a protein binding site. Although putative binding sites can be individually validated using complementary strategies, comprehensive, genome-wide identification of high confidence peaks constitutes a major challenge for ChIP-chip studies.

Several methods have been developed to detect peak regions [3,5-13]. Cawley et al. [3] and Keles et al. [9] applied the Wilcoxon rank sum test and t-test, respectively, to generate test-statistics for sliding windows. Cawley et al. used a fixed p-value cutoff to select peak regions. Whereas Keles et al. employed the Benjamini and Hochberg step-up procedure [14] to control false discovery rate (FDR). In addition to the requirement for experimental replicates, Gottardo et al. [13] identified the absence of powerful multiple testing adjustment methods as a limitation of these methods. Li et al. [7] proposed a hidden Markov model (HMM) approach to identify peak regions, assuming model parameters could be estimated from previous experiments. Ji et al.[6] used a modified t-statistic with a more robust estimate of variance to measure probe-level binding signal, then used either moving window averaging or HMM to estimate window-level binding signal, and finally estimated local false discovery rate (lfdr) of each peak region [15]. Estimation of lfdr requires dissection of the mixture distribution of ChIP-chip signals, which includes the distribution of ChIP enriched signals (or peak signals) and the background (null) distribution. Ji et al.[6] estimated the mixture distribution by unbalanced mixture subtraction, which requires additional information to construct the unbalanced mixtures. Instead of concentrating exclusively on the strengths of binding signals, Zheng et al. [12] identified peaks using both signal strength and signal pattern. Specifically, they modeled the DNA fragmentation process with a Poisson point process and concluded that if the binding signal is transformed to log scale, isolated "peaks" should exhibit a triangular shape allowing development of a double regression method, Mpeak, to identify triangular patterns from ChIP-chip data.

Two recent studies [10,13] have employed Bayesian hierarchical models to identify protein binding sites from ChIP-chip data. A major advantage of Bayesian hierarchical models is that the information across probes can be shared; this is especially important when analyzing a limited number of replicates. However, the difficulty of fitting the complicated Bayesian hierarchical models poses a heavy computational burden. Despite their common characteristics, several attributes distinguish these two approaches. Keles's method[10], HGMM (hierarchical gamma mixture model), adopted a hierarchical gamma-gamma model [16]. HGMM is able to detect peak regions of different sizes. However, its constant coefficient of variation assumption can have an undesired effect in the presence probe outliers [13], and it assumes at most one peak per genomic region, so that the genome has to be partitioned (often arbitrarily) into smaller regions before applying HGMM. Gottardo et. al.'s method [13], BAC (Bayesian Analysis of ChIP-chip), is based on approaches used for gene expression studies [17] with some additional modifications to exploit the spatial dependence between neighboring probes and to improve the robustness for ChIP-chip studies. However, BAC, as it is currently implemented, cannot be applied to a single sample.

In this paper, we propose a mixture model approach to identify peaks from ChIP-chip data. Our method builds on the important observation made by Buck et al. [5] that the signals from ChIP-chip data are not symmetric. When transformed into log scale and represented as a histogram, the signal density often has a heavier right-tail reflective of the presence of true positive signals. It is reasonable to assume that the majority of the left-tail of the signal density arises from background noise, which defines the null distribution. Based on the additional assumption that the null distribution is normal with mean of 0, Buck et al. [5] used negative signals to construct the null distribution and then evaluated the p-values of tested regions. Following Buck et al. [5], we assume that the null distribution is symmetric, but we allow the null distribution to be non-normal and allow its center to deviate from 0. We estimate the local false discovery rate (lfdr) [15] for each peak based on a nonparametric approach to dissect the null distribution (background signals) and alternative distribution (ChIP enriched signals). As pointed by Zheng et al. [12], omitting auto-correlation structure of nearby probes leads to bias in estimating the significance level of each peak. In this study, we adopted the Poisson point process used by Zheng et al. [12] to estimate auto-correlation and incorporate auto-correlation into the lfdr evaluation procedure.

Compared with the existing methods, our method does not rely on potentially restrictive assumptions, such as a normal null distribution [5], or prior knowledge, such as the availability of model parameters[7]. Our major assumption is that the null distribution is symmetric, which can typically be achieved after appropriate normalization (see below). Importantly, our method permits analysis in the absence of replicates, a situation that often arises in exploratory ChIP-chip studies[18]. In addition, our method functions well with abundant peak regions, which is common in the increasing popular epigenetic studies [19,20].

Our method also alleviates the burden of cross array normalization. In large scale studies, a number of arrays are often needed to cover the entire region of interest. Signal differences between arrays may due to technical effects (experimental bias) or relevant biological differences. If prior knowledge implies that there is no systematic biological difference across arrays, it may be more appropriate to combine those arrays prior to the application of peak finding methods. For example, in NimbleScan, the software provided by NimbleGen, the raw data (log ratio) is normalized by subtracting a robust estimate of the sample median. In other words, the data from different arrays are aligned by their medians. However, in practice, it may be difficult to know whether biological differences contribute to systematic differences across arrays. Our method uses the signals derived from one array to identify peaks thereby avoiding the potential problem of cross array normalization. Peaks from different arrays can then be compared by their lfdrs.

In raw data, the null distribution reflecting background noise may not be symmetric and may be heterogeneous depending on the GC-content of the probes [11]. Therefore, within-array data normalization is crucial to the success of our mixture distribution method. Song et al. [11] proposed a normalization method, MA2C (model based 2-color arrays), that normalizes data by assuming the log-intensities of the two channels follow a bivariate distribution with GC-specific means and variances. Song et al. have shown that MA2C standardizes data from different samples more efficiently than other existing methods. Although MA2C works well in many situations, sometime MA2C normalized data still have nonhomogenous null distributions across GC-contents. To overcome this issue, our method uses a Lowess smooth curve to capture the GC-content specific information.

Our mixture model approach is general enough to be applied to one-color arrays (e.g., some Affymetrix tiling arrays), two-color arrays (e.g., some Nimblegen tiling arrays), and high throughput sequencing data. However, since the normalization method pertains to two-color arrays, we focus on its application for two-color arrays. We have implemented our method into an R package, Mixer, which can be downloaded from .

## Methods

### Data normalization

**Figure 1:**
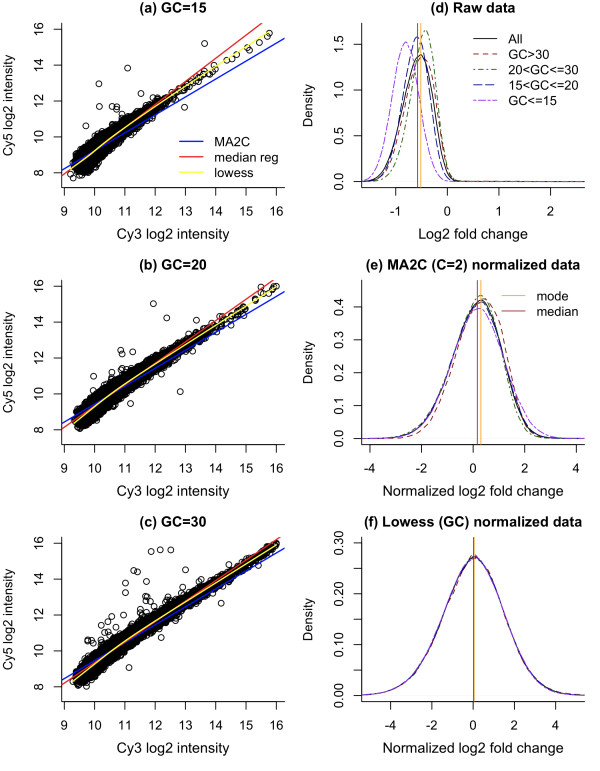
**GC-dependent normalization of one sample**. Scatter plots of log intensities of Cy3 and Cy5 signals (from array GSM254806) based on the number GC base pairs of each 50-mer probe: 15 (a), 20 (b) or 30 (c). Density plots of raw data (d), MA2C (robust, C = 2) normalized data (e) and Lowess normalized data (f). Three curves are overlaid on figures (a)–(c). The blue line depicts the baseline model of MA2C normalization. The red line is fitted by median regression and the yellow line is the Lowess fit. In figures (d)–(f), vertical lines indicate mode and median of all probes. In raw and MA2C normalized data, the mode is bigger than median (d, e), indicating a heavier tail on the left. This unexpected feature usually indicates a problematic array or insufficient normalization.

Let the *x*_2*i *_and *x*_1*i *_be log_2_(Cy5) and log_2_(Cy3) of the *i*-th probe with GC content *k*, and let *μ*_2*k *_and *μ*_1*k *_be the expected value of *x*_2*i *_and *x*_1*i*_, respectively. MA2C normalizes data by calculating



where  and  are robust estimates of *μ*_2*k *_and *μ*_1*k*_, respectively, and  is a robust estimate of the standard deviation of *x*_2*i *_- *x*_1*i *_- ( - ). Considering  = *x*_1*i *_+ ( - )as a predictive value of *x*_2*i *_based on the linear model log_2_(Cy5) = log_2_(Cy3) + *b*_0_, where *b*_0 _is estimated by  - . Then *x*_2*i *_- *x*_1*i *_- ( - ) is the residual from the baseline model log_2_(Cy5) = log_2_(Cy3) + ( - ), and the MA2C normalized value is simply a variance-standardized residual of this linear model with a slope of 1 (see Fig. 6 of Song et al. [11] for an illustration). The underlying assumption of this baseline model is that log_2_(Cy5) - log_2_(Cy3) is constant given GC content. Although this assumption may be sufficient for some samples, the channel differences of log-intensities may depend on the intensities themselves. For example, analyzing previously published array data [21], we found that the channel difference in one array is negative when log_2_(Cy3) and log_2_(Cy5) are small, but approaches 0 as log_2_(Cy3) and log_2_(Cy5) become larger (Figure 1 (a–c)). This variation justifies the use of a fully parameterized linear model: log_2_(Cy5) = b_0 _+ b_1 _× log_2_(Cy3) as the baseline model. Therefore, an improvement over the MA2C normalization would be to assume a linear relation between log_2_(Cy5) and log_2_(Cy3) and estimate both intercept and slope from data in a robust way, for example, using median regression. However, we found that the relation between log_2_(Cy5) and log_2_(Cy3) may be non-linear, and not fully captured by median regression (See Figure 1, and Sup. Figure 1(a–b), Sup. Figure 2(a–b) in Additional file 1). To accommodate non-linear intensity-dependent patterns, we normalized data by Lowess curve fitting conditioning on GC-content. The Lowess normalization is able to account for either linear or non-linear relation and it is robust to outliers. Specifically, given GC-content, let *z*_*i *_= g(*x*_1*i*_) be the Lowess fit (we fit Lowess curve by R function lowess), the normalized log ratio difference is calculated as

**Figure 2:**
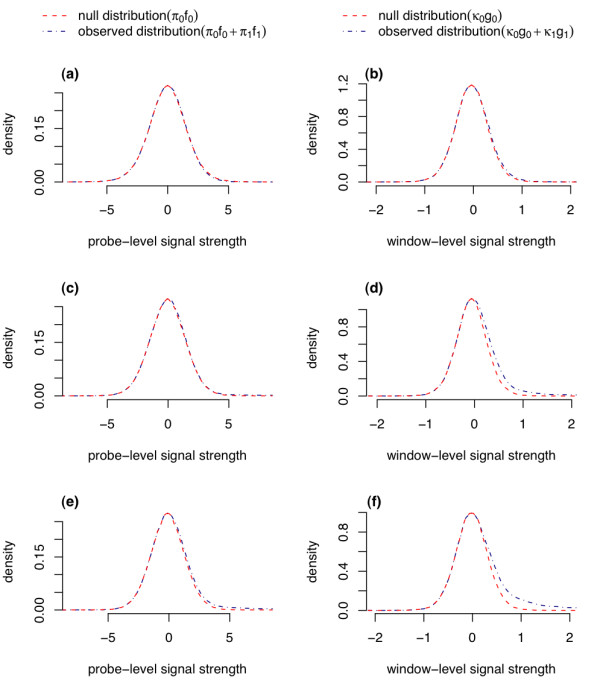
**Dissection of the mixture distribution for probe-level and window-level data**. Mixture distributions for the original spike-in data (a, b), first augmented data with ~4.3% spike-ins (c, d) and the second augmented data with ~10.2% spike-ins (e, f)

(1)

where *M*_*i *_is the median of *x*_2*i *_- *z*_*i*_. We found this Lowess normalization better captured the relationship between signal intensities (See Figure 1(a–c), and Sup. Figure 1(a–b), Sup. Figure 2(a–b) in Additional file 1). Although Lowess normalization has been applied to gene expression microarray data [22-24], to the best of our knowledge, this is its first application to ChIP-chip data.

### Mixture models of ChIP-chip data

ChIP-chip data analysis represents a combined mixture model problem. Observed probe-level data are sampled from the mixture distribution of background signals (null distribution) and ChIP-enriched signals (alternative distribution). In addition, peaks can be detected by moving windows of various lengths. Therefore there are two mixture model problems: one at the probe level and one at the window level. Let *f*_0_(*x*) and *f*_1_(*x*) be the probe level density functions of the null and alternative distributions respectively, and let π_0 _and π_1 _be the corresponding mixture proportions respectively, then the observed probe-level data follows the mixture distribution



We define a window as a fixed length region around a probe. Let the window-level density functions for null and alternative distributions be *g*_0_(*X*) and *g*_1_(*X*) respectively. We use *X *to denote the window level signal strength to distinguish it from the probe level signal strength *x*. Let the corresponding mixture proportions be κ_0 _and κ_1_, then the observed window-level data follows mixture distribution



### Probe-level analysis

We first consider the probe level distribution *f*_*obs*_(*x*) = π_0_*f*_0_(*x*) + π_1_*f*_1_(*x*) Similar to the approach of Buck et al. [5], we utilize lower (but not necessary negative) signals to infer the null distribution *f*_0_(*x*) or *g*_0_(*X*) (described below). We assume that the null distribution is symmetric but place no constraint on the function form or the location of the null distribution.

Let *μ*_0 _be the center of the null distribution, which is approximately the π_0_/2 percentile of the whole distribution assuming that the vast majority of the signals smaller than *μ*_0 _arise from the null distribution. This is a reasonable assumption because most ChIP-enriched signals are higher than the majority of the background signals. Then in order to estimate π_0_, we just need to estimate *μ*_0_. Based on the assumption that the null distribution is symmetric with center *μ*_0_, it is reasonable to assume that *μ*_0 _is the mode of the entire distribution, or one of the two modes if the ChIP-enriched signals also form a mode [25]. Therefore, in order to estimate *μ*_0_, we identify the mode(s) of the observed density *f*_*obs*_(*x*) = π_0_*f*_0_(*x*) + π_1_*f*_1_(*x*)

We first rounded all the probe level signals to a given precision, for example, 0.01 or 0.001 to facilitate subsequent computation. The precision is chosen so that little or no information is lost. We estimate the signal density function by kernel method (R function density with normal kernel) [26,27]. If the estimated density function has two or more modes, we refer to the highest one as the major mode and the others as minor modes. For simplicity, if there is only one mode, we also refer to it as the major mode. A mode cannot be *μ*_0 _if it is bigger than the overall median, otherwise



Specifically, we estimate *μ*_0 _based on the following procedure.

1. If the major mode is smaller than the overall median, we take it as *μ*_0_.

2. If the major mode is bigger than the overall median and there is one and only one minor mode in 20^th ^– 50^th ^percentile of the observed signal (we chose this range for robustness, as explained below), we take the minor mode as *μ*_0_.

3. In all the other situations, we make a conservative estimation of the mode location of the null distribution. Specifically, we iterate all the signal strengths within 20^th ^– 50^th ^percentile (again, we chose this range for robustness, as explained below) and choose the greatest one so that the estimated null distribution is below the overall distribution, i.e., π_0_*f*_0_(*x*) ≤ *f*_*obs*_(*x*) In practice, if such a conservative estimation has to be made, the resulting lfdr is an upper bound instead of an unbiased estimation of actual lfdr.

The major mode can be simply identified as the point with the highest density estimation. The minor mode can be identified as the point where the corresponding 1^st ^derivative of the density function is 0 and the 2^nd ^derivative is negative. We estimate the 1^st ^and 2^nd ^derivatives of the density function by Savitzky-Golay smoothing filters [28-30]. Because there are fewer observations at the tails of a density curve, the kernel estimations there may have bigger variations. This variation could result in "small" modes at the tails that happen by chance. In order to avoid these potentially artifactual modes, we assume *μ*_0 _is within 20^th ^– 50^th ^percentile of the observed signal, which is equivalent to assuming the proportion of null signals is between 40% and 100%. This range is wide enough to accommodate the vast majority of the ChIP experiments. For experiments with even smaller proportions of null signals, pattern reorganization methods that capture ChIP-enriched signals in segments may be more appropriate [31].

After identifying the mode of the null distribution (*μ*_0_), hence π_0_, we take all the data points smaller than *μ*_0_, denoted as D1, all the data points equal to *μ*_0_, denoted as D2, and all the data points generated by flipping D1 around *μ*_0_, denoted as D3, merge them together (i.e., D = {D1, D2, D3}) to estimate the null distribution *f*_0_(*x*) by kernel method (R function density with normal kernel) [26,27]. Finally the probe level lfdr, i.e., the posterior probability that one probe level signal arises from *f*_0_(*x*) is

(2)

where *p*_0_(*x*) indicates the probability that *x *is from the null distribution. In practice, kernel estimation of density functions may be unreliable at the tail area, due to limited number of observations. As a result, the lfdr estimates fluctuate. To circumvent this problem, we order those *x *where the lfdr is evaluated in ascending order x_(1) _≤ x_(2) _≤ ... ≤ x_(m) _and update *p*_0_(*x*_(*i*)_) by



Therefore the estimation of *p*_0_(*x*) is smoothed and decreases or remain the same as *x *increases. A similar strategy has been used to define q-value from FDR estimates[32].

### Window-level analysis

The window-level signal strength *X*, which can be defined as mean or median (or other robust estimations, for example, those used in [11]), is a function of window size and the probe-level signals within the window. In this study, we assume the window size is pre-determined. Let the probe-level signals within one window be *x*_1_, *x*_2_,..., *x*_n_, we calculate *X *as

(3)

where  is the average of probe-level signals and  is the standard error of  under null distribution. In other words, *X *measures the distance between  and *μ*_0_, in terms of the standard error , which is generally bigger than  because there are auto-correlations between nearby probes even for background signals. We estimate  by

(4)

Because we estimate  under null distribution,  depends only on the number of probes in the window and the distances between them, but not the particular probe level signals. This estimation in equation (4) has the same form as the one used by Zheng et al. [12]. However, based on the underlying assumption that the vast majority of the signals are from the null distribution, Zheng et al. used all the data below a threshold to estimate both var(*x*) and corr(*x*_*i*_, *x*_*j*_). In order to accommodate a relatively large proportion of ChIP-enriched signals, we use different approaches to estimate var(*x*) and corr(*x*_*i*_, *x*_*j*_). Specifically, we estimate var(*x*) using the data D = {D1, D2, D3} and estimate corr(*x*_*i*_, *x*_*j*_) as follows. We model the signal strength at probe *j *by

(5)

where *ω*_*ij *_is the probability that there is no break up of the DNA sequence between probe *i *and *j*, and *e*_*ij *_indicates the signal strength at probe *j *due to the DNA segments not harboring probe *i*. *x*_*i *_and *x*_*j *_are measured based on a large number of sequence segments bound to the probe *i *and *j*, respectively. Equation (5) can be understood as a summation of the contributions from all the sequence segments captured by probe *j *from an expectation perspective. Since *e*_*ij *_is independent with *x*_*i*_,

(6)

Because we are modeling the correlation structures in the background signals, var(*x*_*i*_) = var(*x*_*j*_) = var(*x*), hence corr(*x*_*i*_, *x*_*j*_) = *ω*_*ij*_. In order to estimate *ω*_*ij*_, we modeled the sonication process by Poisson point process [12]. Suppose, on average there is one break up of DNA sequence per *k *bp, the incident rate in the Poisson point process is *λ *= 1/*k*, and *ω*_*ij *_= exp(-*λ*d_*ij*_), where d_*ij *_indicates the distance between probe *i *and *j*. Therefore given the parameter *λ *(or equivalently *k*), we can estimate *ω*_*ij*_, hence corr(*x*_*i*_, *x*_*j*_), and then we can calculate the window-level statistics *X*. Usually, the parameter *λ *(or *k*) can be obtained from the experimental setting for the DNA sonication process. For sequencing studies, *ω*_*ij *_can be simply estimated from the distributions of sequence fragment lengths [33].

Next, the window level mixture distribution *g*_obs_(*X*) = κ_0_*g*_0_(*X*) + κ_1_*g*_1_(*X*) can be dissected similarly to the analysis of the probe level data. Finally, the window level lfdr, i.e., the posterior probability that one window-level statistics *X *is from the null distribution is

(7)

where *q*_0_(*X*) indicates the probability that *X *is from the null distribution. Similarly to the probe-level analysis, we smooth the lfdr by updating *q*_0_(*X*_(*i*)_) as



Here *X*_(1) _≤ *X*_(2)... _≤ *X*_(*w*) _are the window-level signals where the lfdr are evaluated.

### Peak Identification

After probe-level and window-level analyses, we identify peaks by the following steps. First, "peak windows" with elevated signal strengths are identified using a window-level lfdr cutoff, e.g., lfdr ≤ 0.20. Second, overlapped "peak windows" are separated into discrete peak regions. Third, each resulting peak region is evaluated by further restriction on the number of probes within it and the signal strengths of those probes. A typical rule could be "a peak region should harbor at least 5 probes", or "a peak region should harbor at least 3 probes with probe level lfdr ≤ 0.2". The third step is optional but recommended since "isolated peaks" composed of only one or two probes are unlikely to represent true sites of protein-DNA interactions. Similar rules have been used in other ChIP-chip data analysis methods [6,12].

## Results

We compared the results of our peak detection strategy with other published algorithms using three datasets. We focused on two common conditions that were typically not evaluated during the development of the existing peak detection algorithms: the absence of experimental replicates and the presence of abundant peak regions.

### Spike-in Data

We initially evaluated our method using the data set from a recent spike-in study [21]. In this benchmark study comparing ChIP-chip conditions, human genomic DNA was combined with defined cloned regions ("spike-ins") over a wide range of concentrations to reflect the enrichment ratios often observed in ChIP experiments. The use of an experimental spike-in data set allows definitive knowledge of the regions that are enriched. Although multiple tiling array designs were tested, since the current implementation of our normalization method is for two-color arrays, we analyzed the data generated from seven NimbleGen arrays. The original data in "pair" format, which includes signals from both Cy3 and Cy5 channels, were downloaded from NCBI GEO database. Four arrays (GEO sample accession number: GSM254930, GSM254971, GSM254972, GSM254973) were hybridized to DNA spiked with specific unamplified fragments. The other three arrays (GSM254805, GSM254806, GSM254807) were hybridized to DNA spiked with fragments that had been amplified. Each array harbors 385,149 probes spanning 44 ENCODE-selected regions[34]. 100 or 98 regions were spike-in with unamplified and amplified DNA, respectively, at various concentrations from 1.25 fold to more than 100 fold. A complete description of these data can be found in Johnson et al. [21].

In the original data, the peak regions were sparse (covering ~0.2% of the total number of probes). We simulated data with increasingly abundant peak regions by replacing the signals from non-spike-in regions with the signals from spike-in regions. To better mimic the original data and more faithfully replicate the flanking contexts, we replicated each spike-in region (450–550 bp) including 500 bp on either side (or to the boundaries of the corresponding ENCODE regions) as a unit, which we refer to as a peak-containing region. Lengths of such peak-containing regions vary from 1,172 bp to 1,550 bp, with median of 1,496 bp. We split the remaining non-peak-containing regions into 18,531 segments of 1,600 bp. We then used the peak-containing regions to replace (fractions of the same lengths of) randomly selected non-peak-containing segments. In the first augmented data set, we replicated each peak-containing region 20 times, resulting in 2,100/2,058 peak-containing regions (covering ~4.3% of the total number of probes) in the unamplified/amplified DNA samples, respectively. In the second augmented data set, we replicated each peak-containing region 50 times, resulting in 5,100/4,998 peak-containing regions (covering ~10.2% of the total number of probes) in the unamplified/amplified DNA samples, respectively.

### Analysis of Spike-in Data

Using the native and augmented spike-in data, we compared the efficacy of our peak detection method, which we named Mixer, with three other methods: MA2C, TileMap, and HGMM. These methods were selected because they are frequently used and/or they also aim to dissect the mixture distributions of ChIP-chip data. BAC by Gottardo et al. [13] was not compared as it requires experimental replicates. Mpeak by Zheng et al. [12] was also not compared because Mpeak assumes that the peaks have triangular shapes. However, the signals from spike-in regions exhibit rectangular patterns.

We used the Java version of MA2C software with the default normalization option ("robust with C = 2"). Other options led to similar or inferior results (data not shown). After normalization, the median was used by MA2C to identify peak regions with a bandwidth (half-width of the sliding window) of 300 bp and at least 5 probes per peak region. A bandwidth of 300 bp was chosen based on the lengths of the spike-in regions. Other bandwidths (500 bp or 200 bp) produced inferior results (data not shown).

For the implementation of Mixer, as with MA2C, we used "half-width of the sliding window of 300 bp with at least 5 probes" as the criteria to select peak regions. We set the average sonicated sequence length as 1000 bp (i.e., *λ *= 1/1000) to estimate the correlation between nearby probes. Substitution of values from 500 bp to 1500 bp did not significantly change the results. In order to demonstrate the difference between Lowess and MA2C normalization, we tested Mixer with data normalized by both methods.

We employed CisGenome[35] for TileMap calculation. Log_2 _transformed data were pre-normalized using the quantile normalization option in CisGenome. TileMap summarizes window-level signals by either moving average or HMM. The significance of each peak is measured by an lfdr estimated from unbalanced mixture subtraction (UMS). We used HMM because it yields superior results in terms of higher power given an lfdr cutoff. Two parameters (p and q) must be provided to UMS to enable selection of probes (with percentiles greater than 100q-th and less than 100p-th) from the overall distribution to construct the null/alternative distributions. We used either the default values (p = 0.01 and q = 0.05) or adjusted values based on the knowledge of true proportion of spike-in signals. Specifically, we set p = 0.002 and q = 0.02 for the original data with ~0.2% of spike-in probes; p = 0.03 and q = 0.08 when ~4.3% of the probes are from spike-ins; p = 0.08 and q = 0.13 when ~10.2% of the probes are from spike-ins.

The R package R/HGMM was used for HGMM calculation. HGMM can take into account a distribution of peak sizes. We generated this distribution based on the actual lengths of the spike-in regions. In most experiments, however, this information can only be estimated. Raw data (PM measure from pair file) were log_2 _transformed and normalized using the preprocess function of R/HGMM before applying the HGMM function.

We examined the influence of the proportion of null signals on Mixer's performance. Figure 2 shows the estimated densities of probe and window-level signals from the original and two simulated dataset from one array. As the number of spike-in regions increases, the right tail of the window-level signal density becomes heavier. The increased signal density enhances accuracy and robustness to dissect the mixture distribution. Similar patterns were also observed for other arrays.

We then evaluated Mixer, MA2C, TileMap and HGMM using the spike-in data. First, given a fixed cutoff of either FDR ≤ 0.20 (for MA2C) or lfdr ≤ 0.20 (for the other methods), we compared the power and actual FDR of these methods (Tables 1, 2 and 3). The discovery of a peak region was counted as a true discovery (or a true positive) if its center was within a spike-in region; otherwise it was counted as a false discovery. Although an alternative comparison would examine the top *K *peaks identified by different methods, we based our comparison on fixed lfdr/FDR. This approach is more relevant since the number of binding sites is typically unknown.

**Table 1:** Comparison of different methods for the original data set

	**Mixer** **(Lowess)**		**Mixer** **(MA2C)**		**MA2C**		**TileMap** **(p = 0.01, q = 0.05)**		**TileMap** **(p = 0.002, q = 0.02)**		**HGMM**	
**Sample**	**D**	**FDR**	**D**	**FDR**	**D**	**FDR**	**D**	**FDR**	**D**	**FDR**	**D**	**FDR**
GSM254930	108	0.28	503	0.84	241	0.66	85	0.08	84	0.07	111	0.20
GSM254971	100	0.28	113	0.37	227	0.64	86	0.09	85	0.09	N/A	N/A
GSM254972	98	0.29	195	0.61	178	0.53	84	0.07	88	0.08	N/A	N/A
GSM254973	98	0.24	92	0.23	146	0.45	71	0.07	73	0.07	N/A	N/A

GSM254805	66	0.20	153	0.56	116	0.43	81	0.22	52	0.09	N/A	N/A
GSM254806	89	0.19	184	0.61	85	0.19	236	0.66	143	0.42	89	0.18
GSM254807	97	0.24	102	0.26	100	0.21	76	0.08	91	0.13	123	0.32

The first four samples, GSM254930, GSM254971, GSM254972, and GSM254973 were spiked with unamplified DNA, while the last three samples GSM254805, GSM254806, and GSM254807 were spiked with amplified DNA. Among the total of 385,149 probes, about 820 (~0.2%) of them are from spike-in regions. We did not obtain results of HGMM for some arrays (N/A) due to failure of function HGMM.

**Table 2:** Comparison of different methods for the simulated data set with 2,100/2,058 spike-in regions for unamplified/amplified samples, respectively

	**Mixer** **(Lowess)**		**Mixer** **(MA2C)**		**MA2C**		**TileMap** **(p = 0.01, q = 0.05)**		**TileMap** **(p = 0.03, q = 0.08)**		**HGMM**	
**Sample**	**D**	**FDR**	**D**	**FDR**	**D**	**FDR**	**D**	**FDR**	**D**	**FDR**	**D**	**FDR**
GSM254930	2159	0.23	1694	0.17	2219	0.28	1475	0.004	1619	0.004	1605	0.03
GSM254971	1965	0.21	2033	0.22	2187	0.30	1395	0.003	1578	0.006	1577	0.03
GSM254972	2015	0.19	2226	0.27	2151	0.27	1553	0.003	1713	0.009	1553	0.03
GSM254973	1575	0.14	1929	0.19	2094	0.28	1334	0.004	1504	0.008	1520	0.02

GSM254805	1982	0.30	1764	0.24	1671	0.27	1034	0.013	1140	0.019	939	0.03
GSM254806	2180	0.27	2344	0.33	1910	0.23	1404	0.008	1687	0.027	1372	0.03
GSM254807	1495	0.14	1926	0.18	2034	0.27	1486	0.003	1655	0.009	1519	0.03

See main text for the simulation methods. Approximately 4.3% of the probes are from spike-in regions.

**Table 3:** Comparison of different methods for the simulated data set with 5,100/4,998 spike-in regions for unamplified/amplified samples, respectively

	**Mixer** **(Lowess)**		**Mixer** **(MA2C)**		**MA2C**		**TileMap** **(p = 0.01, q = 0.05)**		**TileMap** **(p = 0.08, q = 0.13)**		**HGMM**	
**Sample**	**D**	**FDR**	**D**	**FDR**	**D**	**FDR**	**D**	**FDR**	**D**	**FDR**	**D**	**FDR**
GSM254930	4359	0.16	5753	0.28	4829	0.19	2775	0.001	3872	0.003	3707	0.02
GSM254971	4969	0.23	5110	0.23	4697	0.22	2758	0.001	3682	0.005	3615	0.02
GSM254972	5135	0.22	3957	0.19	4738	0.19	2978	0.001	4114	0.011	3558	0.03
GSM254973	4714	0.18	4795	0.20	4560	0.20	2695	0.001	3534	0.003	3493	0.02

GSM254805	4537	0.25	4784	0.27	3860	0.22	1946	0.003	2744	0.022	2237	0.03
GSM254806	4878	0.21	5826	0.32	4284	0.17	2672	0.0004	3924	0.022	3085	0.03
GSM254807	4957	0.21	5157	0.24	4569	0.20	2487	0.0004	3802	0.003	3508	0.02

See main text for the simulation methods. About 10.2% of the probes are from spike-in regions.

We compared the results of Mixer after data normalization by Lowess or by MA2C. For the original data when the spike-in regions are sparse, in general, Mixer performs much better with Lowess normalization than with MA2C normalization. Mixer with MA2C normalization often includes many false discoveries resulting in a high FDR (see Table 1). As spike-in regions become more abundant, the normalization method makes less difference (Table 2, 3). Dissection of the mixture distribution becomes easier with additional data to estimate the alternative distribution, which may overcome the differences attributable to the normalization methods.

We then compared the performance of the peak detection algorithms on the original and augmented data sets. HGMM was computationally intensive, requiring more than 10 hours to analyze one array. In contrast, the other methods we tested completed the analysis of a single array in less than 10 minutes. With the original data, (i.e., no replicates and a small proportion of spike-in regions), HGMM failed for four arrays due to errors in numerical optimization. Although the use of initial values other than the defaults may avoid such errors, we did not explore this due to the high computational cost. In the augmented data sets (with a larger proportion of spike-in regions), HGMM did not fail for any array. However, HGMM was often over-conservative missing 30–50% of spike-in regions (Table 2, 3).

At the default parameters of p = 0.01 and q = 0.05 (i.e. using the top 1% of the data to estimate alternative distribution and 95% of the data to estimate null distribution), TileMap was over-conservative and had limited power, especially when the proportion of spike-in regions is high. TileMap performed much better when provided appropriate values for parameters p and q based on the true proportion of alternative distribution (Tables 1, 2 and 3). However, in actual applications, the alternative distribution is typically unknown. For example, for amplified DNA samples when there are 4998 (~10.2%) spike-in regions, with lfdr smaller than 0.2, TileMap identifies ~70–80% of the spike-in regions if p = 0.08 and q = 0.13, but only ~60% of the spike-in regions with the default parameters, p = 0.01 and q = 0.05.

**Figure 3:**
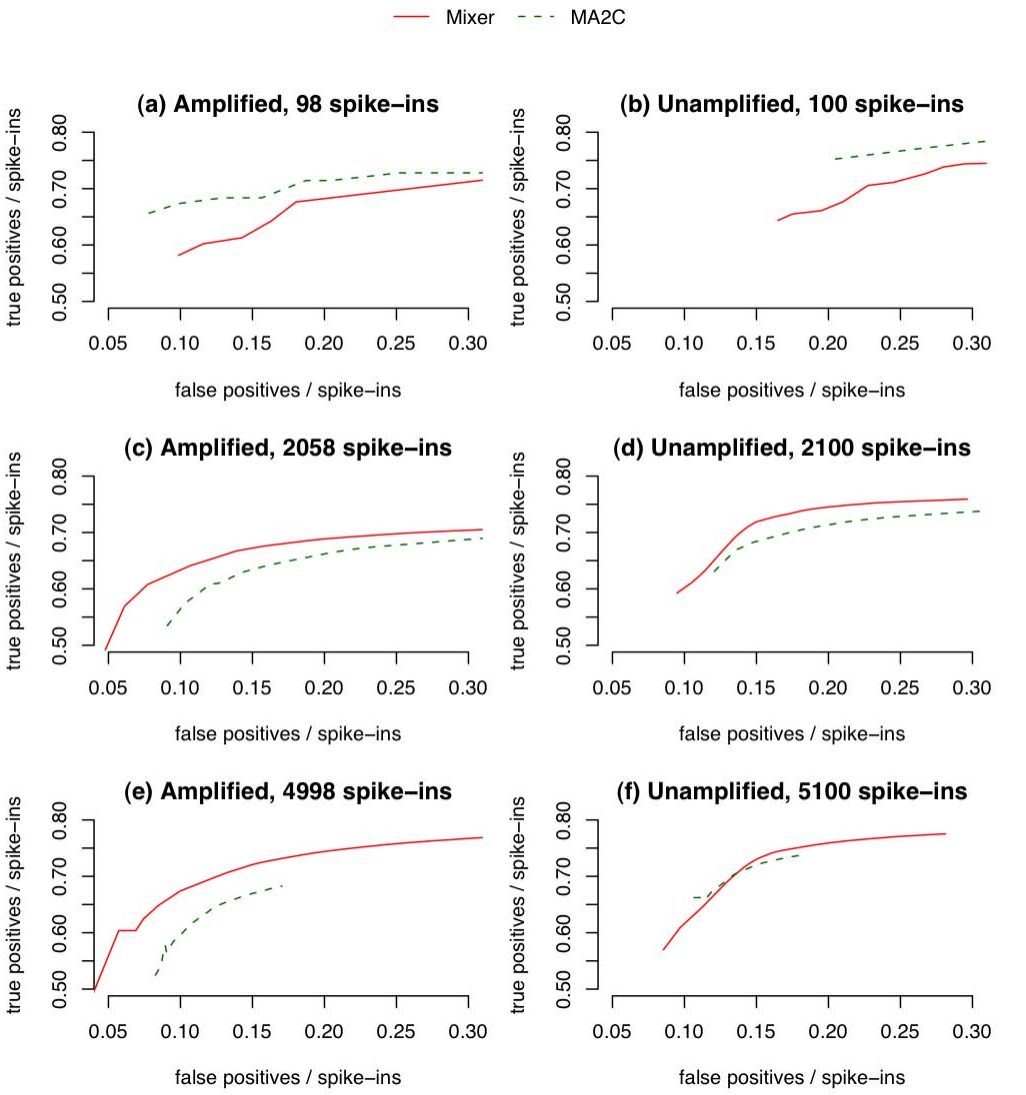
**Comparison of Mixer and MA2C by ROC-like curves**. Peaks were detected by Mixer (with Lowess normalization) or MA2C (with MA2C normalization). Some curves appear to be truncated at the left side because we restrict the cutoff to be FDR or lfdr smaller than 0.5. A larger cutoff is rarely used in practice.

Both Mixer and MA2C have better power than TileMap and HGMM. As shown in Tables 1, 2 and 3, Mixer has lower FDR than MA2C for original data with sparse spike-in regions and has slightly better power than MA2C with abundant spike-in regions. However, a straightforward comparison between Mixer and MA2C is confounded by the fact that, unlike other methods, MA2C provides FDR estimates rather than lfdr estimates. Since lfdr and FDR cutoffs are not directly comparable, we employed ROC (receiver operating characteristic)-like curve to compare Mixer and MA2C (Figure 3). Unlike a typical ROC curve, these ROC-like curves plot (number of true positives)/(number of spike-in clones) on the *Y*-axis against (number of false positives)/(number of spike-in clones) on the *X*-axis in order to accommodate the large number of true negatives in ChIP-chip data, [21]. To simplify the plots, we averaged across samples for amplified/unamplified DNA respectively. FDR and lfdr cutoffs were set between 0.01 to 0.50. Mixer outperformed MA2C when the spike-in regions were abundant (Figure 3). However, when the spike-in regions were sparse, MA2C outperformed Mixer if an appropriate FDR cutoff was chosen.

### Analysis of CTCF-binding Data

We also evaluated our method using the ChIP-chip data from a study of the zinc finger insulator protein CTCF (CCCTC-binding factor) in IMR90 human fibroblast cells[36]. This dataset includes 38 arrays each with about 38,500 50-mer probes tiling the non-repetitive sequences of the human genome in 100 bp resolution. The original pair data (pair data includes the intensities for two channels, Cy5 (CTCF ChIP sample) and Cy3 (input genomic DNA)) were obtained from the Ren laboratory website . Each of the 38 arrays was analyzed separately. The results of different peak-finding algorithms were compared to the results of an independent ChIP-seq based analysis that identified 20,262 CTCF binding sites in human CD4^+ ^T cells [37].

HGMM was not evaluated due to its high computational cost. Model parameters were similar to those described above. For TileMap, window-level signals were summarized by HMM, and the lfdr of each peak region was estimated from unbalanced mixture subtraction (UMS) with default parameters (p = 0.01 and q = 0.05). For MA2C, default options were used to normalize data (robust with C = 2) and summary window-level signals (by median). In Mixer, the average DNA fragment length was set to 1500 bp (T. Kim, personal communication).

Although true CTCF binding sites are unknown, to permit a systematic evaluation of the various peak detection strategies, we compared the peak regions identified by each method with the 20,262 CTCF binding sites reported from a ChIP-seq study by Barski et al. [37]. Since experimental variation will likely result in differences between ChIP-chip and ChIP-seq data, ChIP-seq data serves as a common and independent source for comparison, rather than a perfect standard. A common site was called when the center of the ChIP-chip peak was located within the ChIP-seq peak. Without the knowledge of all true CTCF binding sites we are unable to compare FDRs, as we had done for the spike-in data. Therefore, we examined a fixed number of high confidence peak regions and compared the proportion of overlap. Specifically, we examined the overlap between the ChIP-seq reported sites and 5,000, 10,000, or 20,000 peak regions with the highest confidence (lowest FDR or lfdr) identified by each peak detection algorithm. Peaks identified by Mixer consistently demonstrate a greater overlap with ChIP-seq peaks than those identified by MA2C and TileMap (Table 4).

**Table 4:** Comparison of the peaks identified by Mixer, MA2C, and TileMap with sites identified by ChIP-seq.

**Total Number of peak regions**	**Mixer**		**MA2C**		**TileMap**	
	
	**Peaks**	**Lfdr**	**Peaks**	**FDR**	**Peaks**	**lfdr**
5,000	2974 (59.5%)	0	2421 (48.4%)	0	2090 (41.8%)	≤ 6 × 10^-6^
10,000	5909 (59.1%)	≤ 2.4 × 10^-4^	4840 (48.4%)	0	4049 (40.5%)	≤ 7 × 10^-6^
20,000	8931 (44.7%)	≤ 0.046	8217 (41.1%)	≤ 0.032	7270 (36.4%)	≤ 3.1 × 10^-5^

In each cell, the number of overlapped peak regions and the percentage among the top *k *peak regions are shown, where *k *= 5,000, 10,000, or 20,000.

### Analysis of FAIRE Data

We also compared Mixer, MA2C, and TileMap on array data produced by hybridization of DNA enriched by Formaldehyde-Assisted Isolation of Regulator Elements (FAIRE)[19,38]. Briefly, FAIRE identifies open chromatin regions using organic extraction of formaldehyde crosslinked chromatin. DNA recovered in the aqueous phase is fluorescently labeled and hybridized to arrays. FAIRE typifies the data from epigenetic studies where relevant features are expected to be abundant genome-wide. FAIRE-chip thus provides an appropriate application for Mixer. For this analysis, FAIRE was performed on chromatin isolated from human foreskin fibroblasts and hybridized to a 1% ENCODE tiling array at 38-bp resolution [19].

Four arrays hybridized with FAIRE-selected chromatin were normalized individually. After averaging identical probes across the arrays Mixer was applied. MA2C and TileMap were run using their default options for replicate analysis. Since hypersensitivity to endonucleases is a standard method to identify open chromatin regions, we compared the results with 3,150 open chromatin regions identified by DNase I hypersensitivity-chip in lymphoblastoid cell lines [39,40]. The FAIRE regions identified by each of the three methods share ~40% overlap with DNase sites, indicating similar specificities for the various methods. Since different techniques and different cell lines are compared, this overlap likely represents an underestimate of specificity. However, Mixer offers increased sensitivity as it identifies more peaks (especially those peaks with relatively weaker signals) at the same specificity. At a local FDR (for Mixer or TileMap) or FDR (for MA2C) cutoff of 0.2, Mixer identifies 1137 peaks (42.1% overlap with DNase hypersensitivity sites) whereas MA2C identifies 750 sites (43.3% overlap), and TileMap identifies 1114 sites (40.3% overlap). At a local FDR/FDR cutoff of 0.5, Mixer identifies 1559 peaks (40.3% overlap); MA2C identifies 1175 (39.7% overlap); and TileMap identifies 1202 (39.7% overlap).

A local FDR less than 0.5 is a much more stringent cutoff than FDR less than 0.5. The former means that the highest FDR for any one of the peak regions is 0.5, whereas the latter indicates that the average FDR is 0.5. Averaging the local FDR less than 0.5 results in an estimated FDR for Mixer or TileMap of less than 0.15. Because it uses a less stringent FDR cutoff, MA2C is expected to identify more peaks. The actual identification of fewer peaks by MA2C suggests the introduction of bias by MA2C normalization. To test this hypothesis, we supplied MA2C with Mixer-normalized data and observed a significant improvement of its sensitivity; 1,483 peaks (~40% overlap DNase sites) were identified at FDR less than 0.20, still fewer than the 1,559 peaks identified by Mixer with an estimated FDR of less than 0.15.

## Discussion

We have developed a mixture model approach to dissect the mixture distributions of ChIP-chip data: the null distribution (corresponding to the background signals) and the alternative distribution (corresponding to the ChIP-enriched signals), at both probe and window levels. This approach builds on the method of Buck et al. [5] to estimate null (background) distribution of ChIP-chip signal data and utilizes the Poisson point process assumption proposed by Zheng et al. [12] to model DNA fragmentation. An advance over most existing peak detection strategies, our approach is less dependent on key assumptions and prior knowledge. Our method takes into account the auto-correlation structure of nearby probes, permits a relatively large proportion of ChIP-enriched signals in the mixture distribution, and does not require cross-array normalization. After dissecting the mixture distribution, both probe-level and window-level lfdrs are provided to evaluate the statistical significance of the identified peaks. Using three data set representing widely divergent experimental conditions, we demonstrated that our method performs comparably or better than several representative existing methods, especially when the true peak regions are abundant. Our method also applies Lowess fit data normalization to capture the non-linear relationship between log(Cy3) and log(Cy5) signals from two-color arrays. Mixer emphasizes the identification of abundant short peak regions rather than extended binding regions. We have recently developed a different method to identify broad signal patterns [31].

Despite Mixer's advances, areas for improved performance remain. We smooth the lfdr estimate so that it decreases as probe-level/window-level signals increase. This smoothing strategy avoids major fluctuations of lfdr estimates when observations are limited (e.g. in tail areas). A similar strategy has been used to define q-value from FDR estimates [32]. However, smoothing may lead to under-estimates of the lfdr, especially for small lfdr. To improve the lfdr estimates, both signal strength and signal pattern (for example the "triangle" pattern used by Zheng et al. [12]) could be incorporated, a strategy we are currently evaluating.

The use of high throughput sequencing based chromatin identification (ChIP-seq) has become increasingly common. However, determination of sufficient sequencing depth remains a significant challenge, especially for abundant epigenetic events. ChIP-chip remains a valuable method for pilot experiments and to cross validate results, a particularly appropriate application of Mixer. Mixer could also be adapted to dissect mixture distributions from sequencing data. Tag counts derived from unfractionated input control could model a null distribution [41]. We are currently testing this approach.

## Conclusion

In summary, we have developed a method that combines improved data normalization and peak detection for ChIP-chip studies. Mixer offers several advantages including lfdr determination and enhanced performance when peak regions are abundant, a common scenario for genome-wide studies of chromatin organization and epigenetics [4,19,20].

## Availability and requirements

We have implemented our method in an R package mixer, which can be freely downloaded from . The source code can be redistributed and/or modified under the terms of the GNU General Public License as published by the Free Software Foundation.

## Authors' contributions

All authors have read and approved the final manuscript. WS, IJD and MJB conceived this study. WS implemented the methods and analyzed the data. WS, IJD, MJB and MP wrote the paper.

## Supplementary Material

Additional File 1 **Supplementary Materials for "Improved ChIP-chip analysis by mixture model approach"**. Supplementary results demonstrating different data normalization methods. Click here for file

## References

[B1] Ren B, Robert F, Wyrick JJ, Aparicio O, Jennings EG, Simon I, Zeitlinger J, Schreiber J, Hannett N, Kanin E (2000). Genome-wide location and function of DNA binding proteins. Science.

[B2] Lieb JD, Liu X, Botstein D, Brown PO (2001). Promoter-specific binding of Rap1 revealed by genome-wide maps of protein-DNA association. Nat Genet.

[B3] Cawley S, Bekiranov S, Ng HH, Kapranov P, Sekinger EA, Kampa D, Piccolboni A, Sementchenko V, Cheng J, Williams AJ (2004). Unbiased mapping of transcription factor binding sites along human chromosomes 21 and 22 points to widespread regulation of noncoding RNAs. Cell.

[B4] Kim TH, Barrera LO, Zheng M, Qu C, Singer MA, Richmond TA, Wu Y, Green RD, Ren B (2005). A high-resolution map of active promoters in the human genome. Nature.

[B5] Buck MJ, Nobel AB, Lieb JD (2005). ChIPOTle: a user-friendly tool for the analysis of ChIP-chip data. Genome Biol.

[B6] Ji H, Wong WH (2005). TileMap: create chromosomal map of tiling array hybridizations. Bioinformatics.

[B7] Li W, Meyer CA, Liu XS (2005). A hidden Markov model for analyzing ChIP-chip experiments on genome tiling arrays and its application to p53 binding sequences. Bioinformatics.

[B8] Johnson WE, Li W, Meyer CA, Gottardo R, Carroll JS, Brown M, Liu XS (2006). Model-based analysis of tiling-arrays for ChIP-chip. Proc Natl Acad Sci USA.

[B9] Keles S, Laan MJ van der, Dudoit S, Cawley SE (2006). Multiple testing methods for ChIP-Chip high density oligonucleotide array data. J Comput Biol.

[B10] Keles S (2007). Mixture modeling for genome-wide localization of transcription factors. Biometrics.

[B11] Song JS, Johnson WE, Zhu X, Zhang X, Li W, Manrai AK, Liu JS, Chen R, Liu XS (2007). Model-based Analysis of 2-Color Arrays (MA2C). Genome Biol.

[B12] Zheng M, Barrera LO, Ren B, Wu YN (2007). ChIP-chip: data, model, and analysis. Biometrics.

[B13] Gottardo R, Li W, Johnson WE, Liu XS (2008). A flexible and powerful bayesian hierarchical model for ChIP-Chip experiments. Biometrics.

[B14] Benjamini Y, Hochberg Y (1995). Controlling the false discovery rate: a practical and powerful approach to multiple testing. Journal of the Royal Statistical Society, Ser B.

[B15] Efron B, Tibshirani R, Storey J, Tusher V (2001). Empirical Bayes analysis of a microarray experiment. Journal of the American Statistical Association.

[B16] Newton MA, Noueiry A, Sarkar D, Ahlquist P (2004). Detecting differential gene expression with a semiparametric hierarchical mixture method. Biostatistics.

[B17] Newton MA, Kendziorski CM, Richmond CS, Blattner FR, Tsui KW (2001). On differential variability of expression ratios: improving statistical inference about gene expression changes from microarray data. J Comput Biol.

[B18] Mardis ER (2007). ChIP-seq: welcome to the new frontier. Nat Methods.

[B19] Giresi PG, Kim J, McDaniell RM, Iyer VR, Lieb JD (2007). FAIRE (Formaldehyde-Assisted Isolation of Regulatory Elements) isolates active regulatory elements from human chromatin. Genome Res.

[B20] Wang Z, Zang C, Rosenfeld JA, Schones DE, Barski A, Cuddapah S, Cui K, Roh TY, Peng W, Zhang MQ (2008). Combinatorial patterns of histone acetylations and methylations in the human genome. Nat Genet.

[B21] Johnson DS, Li W, Gordon DB, Bhattacharjee A, Curry B, Ghosh J, Brizuela L, Carroll JS, Brown M, Flicek P (2008). Systematic evaluation of variability in ChIP-chip experiments using predefined DNA targets. Genome Res.

[B22] Berger JA, Hautaniemi S, Jarvinen AK, Edgren H, Mitra SK, Astola J (2004). Optimized LOWESS normalization parameter selection for DNA microarray data. BMC Bioinformatics.

[B23] Workman C, Jensen LJ, Jarmer H, Berka R, Gautier L, Nielser HB, Saxild HH, Nielsen C, Brunak S, Knudsen S (2002). A new non-linear normalization method for reducing variability in DNA microarray experiments. Genome Biol.

[B24] Yang YH, Dudoit S, Luu P, Lin DM, Peng V, Ngai J, Speed TP (2002). Normalization for cDNA microarray data: a robust composite method addressing single and multiple slide systematic variation. Nucleic Acids Res.

[B25] Buck MJ, Lieb JD (2004). ChIP-chip: considerations for the design, analysis, and application of genome-wide chromatin immunoprecipitation experiments. Genomics.

[B26] R Development Core Team (2007). R: A language and environment for statistical computing.

[B27] Silverman BW (1986). Density Estimation.

[B28] Savitzky A, Golay MJE (1964). Smoothing and Differentiation of Data by Simplified Least Squares Procedures. Anal Chem.

[B29] Steinier J, Termonia Y, Deltour J (1972). Smoothing and differentiation of data by simplified least square procedure. Anal Chem.

[B30] Press WH, Flannery BP, Teukolsky SA, Vetterling WT (1992). Numerical Recipes in C, The Art of Scientific Computing.

[B31] Sun W, Xie W, Xu F, Grunstein M, Li K-C (2009). Dissect nucleosome free regions by a segmental semi-Markov model. PLoS ONE.

[B32] Storey JD, Tibshirani R (2003). Statistical significance for genomewide studies. Proc Natl Acad Sci USA.

[B33] Fejes AP, Robertson G, Bilenky M, Varhol R, Bainbridge M, Jones SJ (2008). FindPeaks 3.1: a tool for identifying areas of enrichment from massively parallel short-read sequencing technology. Bioinformatics.

[B34] (2004). The ENCODE (ENCyclopedia Of DNA Elements) Project. Science.

[B35] Ji H, Jiang H, Ma W, Johnson DS, Myers RM, Wong WH (2008). An integrated software system for analyzing ChIP-chip and ChIP-seq data. Nat Biotechnol.

[B36] Kim TH, Abdullaev ZK, Smith AD, Ching KA, Loukinov DI, Green RD, Zhang MQ, Lobanenkov VV, Ren B (2007). Analysis of the vertebrate insulator protein CTCF-binding sites in the human genome. Cell.

[B37] Barski A, Cuddapah S, Cui K, Roh T-Y, Schones DE, Wang Z, Wei G, Chepelev I, Zhao K (2007). High-Resolution Profiling of Histone Methylations in the Human Genome. Cell.

[B38] Giresi PG, Lieb JD (2009). Isolation of active regulatory elements from eukaryotic chromatin using FAIRE (Formaldehyde Assisted Isolation of Regulatory Elements). Methods.

[B39] Crawford GE, Davis S, Scacheri PC, Renaud G, Halawi MJ, Erdos MR, Green R, Meltzer PS, Wolfsberg TG, Collins FS (2006). DNase-chip: a high-resolution method to identify DNase I hypersensitive sites using tiled microarrays. Nat Methods.

[B40] Sabo PJ, Kuehn MS, Thurman R, Johnson BE, Johnson EM, Cao H, Yu M, Rosenzweig E, Goldy J, Haydock A (2006). Genome-scale mapping of DNase I sensitivity in vivo using tiling DNA microarrays. Nat Methods.

[B41] Rozowsky J, Euskirchen G, Auerbach RK, Zhang ZD, Gibson T, Bjornson R, Carriero N, Snyder M, Gerstein MB (2009). PeakSeq enables systematic scoring of ChIP-seq experiments relative to controls. Nat Biotechnol.

