# Supplementary material for: Improved ChIP-chip analysis by a mixture model approach
Source: BMC Bioinformatics. 2009 Jun 7;10:173. doi: 10.1186/1471-2105-10-173 (PMC2700807; doi:10.1186/1471-2105-10-173)

Supplementary Materials for "Improved ChIP-chip analysis by mixture model approach"

Wei Sun[1,5], Michael J Buck[2], Mukund Patel[3], and Ian J Davis[3,4,6]

[1] Department of Biostatistics, Carolina Center for Genome Sciences, University of North Carolina at Chapel Hill, NC

[2] Department of Biochemistry, Center of Excellence in Bioinformatics and Life Sciences, State University of New York at Buffalo, NY

[3] Department of Genetics, University of North Carolina at Chapel Hill, NC

[4] Department of Pediatrics, Lineberger Comprehensive Cancer Center, University of North Carolina at Chapel Hill, NC

[5] To whom computational biology questions should be addressed, wsun@bios.unc.edu

[6] To whom biological questions should be addressed, ian_davis@med.unc.edu

Supplementary Figure 1 – Normalization of array 41149 of Kim et al. (2007)

Scatter plots of log intensities of Cy3 and Cy5 signals based on the number GC base pairs of each 50-mer probe: 15 (a), 20 (b) or 30 (c). Density plots of raw data (d), MA2C (robust, C=2) normalized data (e) and Lowess normalized data (f). Three curves are overlaid on figures (a)-(c). The blue line depicts the baseline for MA2C normalization. The red line is fitted by median regression and the yellow line is the Lowess fit. In figures (d)-(f), vertical lines indicate mode and median of all probes. In raw and MA2C normalized data, the mode is bigger than median (d, e), indicating a heavier tail on the left. This unexpected feature usually indicates a problematic array or insufficient normalization.

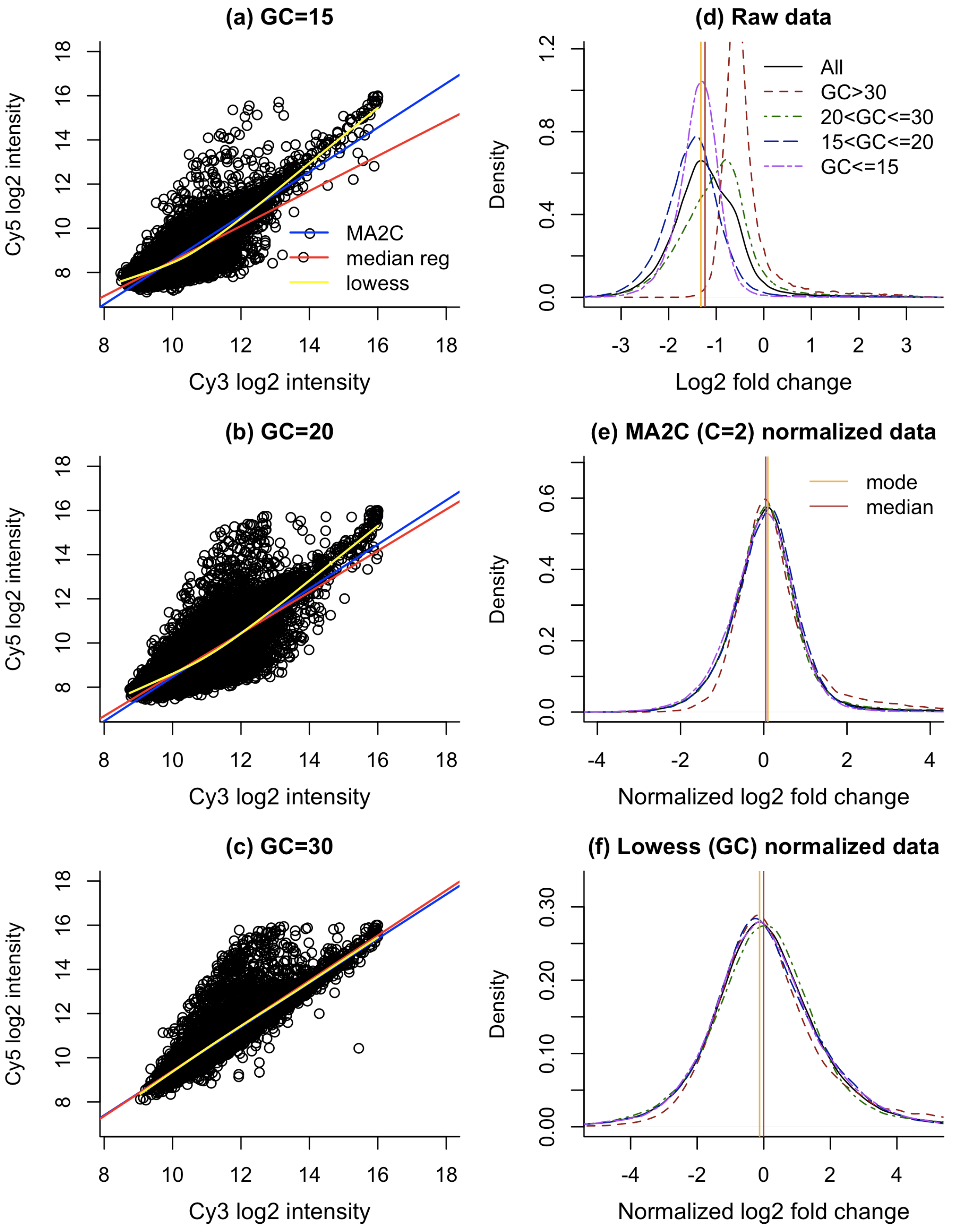

Supplementary Figure 2 – Normalization of array 41946 of Kim et al. (2007)

Similar to Supplementary Figure 1, this figure show the scatter plots of log intensities of Cy3 and Cy5 signals based on the number GC base pairs, and densities of raw data, MA2C normalized data, and Lowess normalized data. Both Supplementary Figure 1 and Supplementary Figure 2 show that the relation between Cy3 and Cy5 signals could be non-linear, which can be well captured by Lowess fit, but cannot be fully captured by MA2C normalization or by median regression.

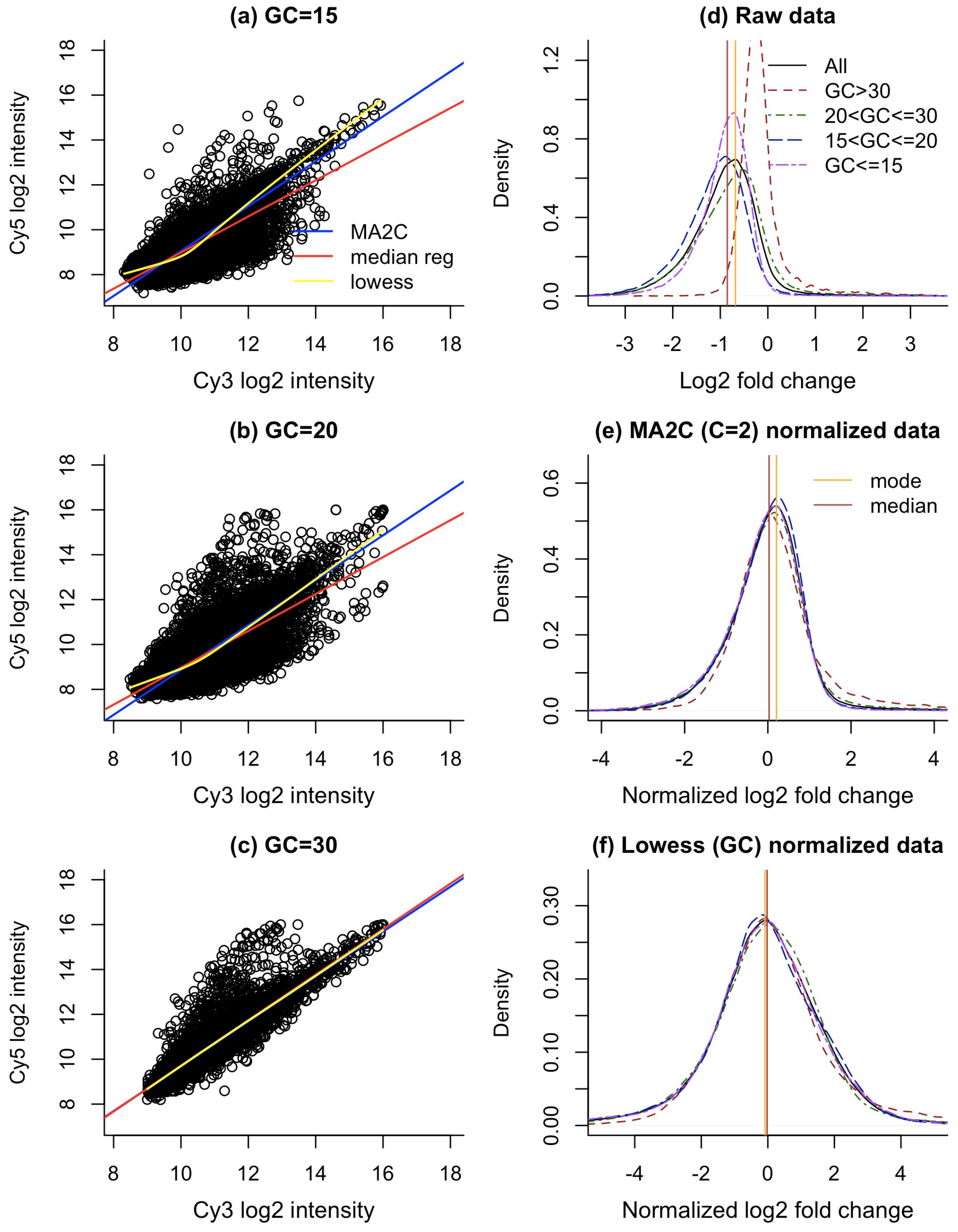

Supplement: Additional File 1 — Supplementary Materials for "Improved ChIP-chip analysis by mixture model approach". Supplementary results demonstrating different data normalization methods. [file 1471-2105-10-173-S1.pdf]
